# Usability and feasibility of multimedia interventions for engaging patients in their care in the context of acute recovery: A narrative review

**DOI:** 10.1111/hex.12957

**Published:** 2019-09-05

**Authors:** Jo McDonall, Anastasia F. Hutchinson, Bernice Redley, Patricia M. Livingston, Mari Botti

**Affiliations:** ^1^ Faculty of Health, School of Nursing and Midwifery, Centre for Quality and Patient Safety Research Deakin University Geelong Vic Australia

**Keywords:** acute care, interventions, multimedia, patient participation

## Abstract

**Purpose:**

The purpose of this narrative review was to examine the usability and feasibility of multimedia intervention as a platform to enable patient participation in the context of acute recovery and to discover what outcomes have been measured.

**Data sources:**

A narrative review of primary research articles identified through a search of four electronic databases (MEDLINE, CINAHL, EMBASE and PsycInfo) identified peer‐reviewed research evidence published in English language with no limitation placed on time period or publication type. Two authors independently assessed articles for inclusion. From the 277 articles identified through the search, 10 papers reporting the outcomes of seven studies were included in this review.

**Review methods:**

Articles were independently assessed for quality and relevance by two authors. The most appropriate method for data synthesis for this review was a narrative synthesis.

**Results:**

From the narrative synthesis of study outcomes, two findings emerged as follows: (a) multimedia interventions are feasible and usable in the context of acute care, and (b) multimedia interventions can improve patients’ perception of care‐related knowledge. Identified gaps included a lack of evidence in relation to the effect of interventions on enhancing patients’ ability to participate in their care and the impact on patients’ health‐related outcomes.

**Conclusions:**

In conclusion, there is some evidence of the feasibility and usability of multimedia interventions in acute care. That is, patients can use these types of platforms in this context and are satisfied with doing so. Multimedia platforms have a role in the delivery of information for patients during acute recovery; however, the effectiveness of these platforms to engage and enhance patients’ capability to participate in their recovery and the impact on outcomes needs to be rigorously evaluated.

## 1 INTRODUCTION

It is well established that engaging patients in their care produces better health outcomes for patients with chronic illness.^1^, ^2^, ^3^ Emerging evidence suggests participation can enhance patient outcomes in acute care environments, particularly in relation to patient safety^4^, ^5^ and satisfaction.^6^, ^7^, ^8^ Despite the perceived and emerging benefits of promoting patient participation in their health care, there is a notable lack of studies evaluating the effectiveness and sustainability of interventions to promote patient participation in acute health‐care environments. Challenges associated with achieving patient participation in acute care include the higher acuity of illness,^8^, ^9^, ^10^, ^11^ greater complexity in medical treatment regimens,^12^, ^13^ and shorter length of stay compared to other non‐acute environments.^14^, ^15^ These factors may all influence patients’ ability to participate in their care to the level they would prefer, and in a way that may affect their outcomes.

To date, patient participation research in acute care has foci across five areas: (a) preference for participation in care,^16^, ^17^, ^18^ (b) experience of participation,^19^, ^20^, ^21^ (c) participation in decision making,^22^, ^23^, ^24^ (d) participation in safety initiatives to minimize adverse events,^5^, ^25^, ^26^, ^27^ and (e) participation in patient‐clinician communication during transitions of care and discharge planning.^23^, ^28^, ^29^, ^30^

Outcomes of research examining patients’ preferences for participation suggest patients want to be involved in their care, but often feel they do not have the capability or opportunity to do so.^19^, ^21^, ^31^, ^32^, ^33^ The majority of this research has been descriptive aimed to elicit patients’ preferences for participation in acute care. For example, McMurray^32^ interviewed patients to gain their perspectives of participation in shift‐to‐shift, bedside nursing handover. Patients were asked their views about bedside handover including the benefits and limitations, their existing and potential role in handover, the role of family members, and issues related to confidentiality. Findings revealed four major themes.^32^ First, patients valued being recognized as “partners”. Second, patients viewed bedside handover as a chance to correct any mistakes communicated during the interaction. Third, some patients preferred to be passive rather than full engagement in the handover process, and fourth, most patients appreciated the inclusive approach as it facilitated nurse‐patient interactions.^32^ When patients’ actual experience of participation in nursing care was examined, Tobiano et al^19^ found that patients described a power imbalance and expressed feelings that their opportunities for participation were restricted. These findings suggest the opportunity for participation in their care needs to be explicit to patients, and facilitated by clinicians so that it is clear that their participation is welcomed and expected, to support patients’ confidence and motivation to engage in the process.^19^ The question therefore is how do we as nurses engage patients in their care at the level that is desired by individual patients.

Patient participation specifically in decision making has been explored in a descriptive study by Kolovos^22^ that found that although patients were involved in planning and implementation of nursing care their level of participation was moderate. In addition, the results provided evidence that patient education correlated with the degree of participation, highlighting the importance of patients understanding exactly where and how they can participate in their care and recovery. Therefore, how patients receive this information to enable participation, in the context of acute recovery, is an important consideration.

Outcomes of a cluster randomized controlled trial testing a complex, multiple component intervention to reduce falls and adverse events (pressure injury, urinary tract infections) showed a reduction in falls and adverse events.^34^ The intervention was designed to involve patients and families by providing written and verbal information related specifically to each patient's identified risks. Although successful, the intervention was detailed and complex to apply, resource‐intensive and dependent on several health disciplines working together, raising questions about its sustainability over time. Further, it was difficult to disentangle the role that patient participation versus staff engagement in risk‐reduction strategies played in achieving the reported outcomes. This raises the question of sustainability of interventions over time. If we are to introduce interventions to enable patients to engage, they must be sustainable without the control conditions of a study.

O'Leary et al^7^ tested a ‘patient‐centred bedside rounds’ intervention in a cluster randomized controlled trial. The intervention involved a multidisciplinary team, using a structured communication tool designed to be used at the bedside. The tool was based on a communication framework where clinicians were given direct instructions, for example, introduce yourself to the patient, update patients’ care team on the white board, review report from previous shift, perform safety checklist and plan discharge. The hypothesis was that patients who were more informed of their care plan and engaged with the members of their health‐care team would be more activated. The authors reported that patient‐centred bedside rounds were only partially implemented (54% of patient handovers) and that there was no difference between groups in patient preference for participation, patient activation or satisfaction with care. Due to the poor uptake of the intervention, the authors questioned whether clinicians valued the inclusion of patients in the transition process.^7^ Gonzalo et al^35^ also found that ‘inter‐professional bedside rounds’ occurred only 64% of the time and were more likely to occur with younger doctors and during periods of lower workload. Strategies to enhance patient confidence to participate in their care and recovery during an acute care admission, where time constraints and other factors present particular challenges, are not well understood.

Typically, interventions tested to engage patients in acute care have included written paper‐based materials,^36^ visual materials such as posters,^37^ video instructions^25^, ^38^ and illness specific tailored education programmes such as falls prevention^39^ or pain management initiatives.^36^, ^39^, ^40^ These methods are not interactive nor typically tailor‐made to patients’ specific educational needs or literacy level. Resources to support patient participation in their care following surgery need to be procedure‐specific but also provide patients with clear guidance about how and when they can participate in their recovery. To enable participation in acute care, patients need to be provided with timely information, relevant to their stage of recovery that can be used to support and encourage their participation.

Rapid advances in information technology and multimedia techniques in the past decade provide novel and unique opportunities for innovative approaches to overcome barriers to patient participation in their care in acute care settings. For example, use of multimedia platforms to provide patient information and education has increased significantly over the past decade. Multimedia tools have being successfully used in a wide range of health situations including preparing patients for specific procedures or surgery by providing education pre‐operatively or to gain pre‐operative consent^41^, ^42^, ^43^, ^44^, ^45^, ^46^, ^47^; providing health information for patients to assist them to make informed decisions regarding treatment^48^, ^49^; presenting information to enable self‐management in chronic illness^50^; increasing knowledge about post‐operative care, for example how to use a patient‐controlled analgesic pump after surgery^51^; and improving patient satisfaction overall.^52^ Two systematic reviews examining the use of multimedia technologies to facilitate the patient education process^53^, ^54^ concluded that these technologies are beneficial in delivering patient education, and value added to the patient education process in terms of increased knowledge, increased confidence in self‐care and ability to participate in decision making.^53^, ^54^ However, evidence for the use of these types of interventions drawn from the chronic illness and ambulatory care settings may not translate to acute care where the barriers and constraints differ. What is less clear is the usability for patients of multimedia interventions during acute recovery from illness or surgery. Further, evidence that multimedia interventions provide patients with the capability to participate and improve patient outcomes is not available.

The purpose of this narrative review was to examine the usability and feasibility of multimedia intervention as a platform to enable patient participation in the context of acute recovery, and to discover what outcomes have been measured as a result of using multimedia.

For the purpose of this review, *multimedia* was defined as a tool that uses animation, sound and text,^55^ *usability* was defined as the degree to which a multimedia intervention is easy to use for patients in the acute care context,^55^ and *feasibility* was defined as the ease or convenience of applying a multimedia intervention.^55^ *Acute care* was defined as a pattern of health care in which patients are treated for brief but severe episode of illness, for example recovery following accident or trauma or during recovery from surgery.^56^

## 2 METHODS

### 2.1 Review questions

A specific mnemonic for qualitative systematic reviews (PICO) was used to develop the question for this review.^57^ Patient (specifically in acute care context), Intervention (multimedia interventions), Comparison (usual care) and Outcome (did the intervention enable patients to participate in their recovery? what outcomes have been measured in acute care? and what is the usability and feasibility of multimedia interventions in acute care?).

The research questions were as follows:

1. Are multimedia interventions effective in engaging patients in their care in the context of acute recovery? and
2. What outcomes have been measured?

### 2.2 Search method

Four electronic databases were searched as follows: MEDLINE, CINAHL, EMBASE and PsycInfo in November, 2015 and repeated in October, 2016 and June 2018. No limitations were placed on the time period or publication type. Three concepts were used to guide the search terms and synonyms used in the strategy: multimedia interventions, *and* acute hospital care *and* patient participation (Table 1). Each database was also searched for relevant subject headings. Google Scholar was used to screen for grey literature, as well as citation searches and reference lists of included studies, and websites of peak bodies. Inclusion and exclusion criteria were developed, reviewed and agreed by the authors (Table 2). This criterion was quite specific and was used to limit the scope of the review.

**Table 1:** Search terms used

Patient OR client OR consumer OR user OR customer OR recipient
AND
Participation OR engagement OR involvement OR collaboration
AND
Interventions OR tools OR multimedia, education
AND
Acute care OR hospitalised OR hospitalised OR inpatient OR hospital OR acute OR post‐operative OR postoperative

**Table 2:** Inclusion and exclusion criteria

Inclusion Criteria	Exclusion Criteria
Adult patients In hospital ‐ specifically acute care clinical setting Multimedia as the intervention tested Must have had a specific aim to enhance patient engagement, involvement or participation	Did not report outcomes from the use of the intervention (ie study protocols, reviews or discussion papers) Did not describe the intervention Was not specifically multimedia or did not incorporate two or more methods (text, sound, graphics) Not written in English language Pre‐admission or outpatient settings (attached to acute hospital however not inpatient acute care)

### 2.3 Data synthesis

Narrative synthesis was deemed most appropriate approach to use as it allows the combination of qualitative, quantitative and multiple design methodologies. Narrative reviews can be performed in different ways and is determined by the research question and types/characteristics of articles included.^58^ A narrative synthesis was undertaken rather than meta‐analysis as there were differences in populations, outcomes and methods used in the studies that would make the average effect across studies futile. The first step of the review involved developing a plan to assessing the studies to be included. The plan was based on the predefined aims and questions for the review. The second step involved a review of the studies by two reviewers (JM and AH) and involved more in‐depth examination of study characteristics (study aim/s, country and setting, intervention, methods and relevant key findings). A review of the findings across all included studies was undertaken to identify themes. This was done independently by the two reviewers who then came together to discuss their findings. If there were any discrepancies that could not be resolved, a third reviewer would be asked; however, this was not required in this instance. Both reviewers agreed on the themes identified. The findings of the studies were summarized in tables based on the predefined questions.

## 3 RESULTS

### 3.1 Study characteristics

**Figure 1:**
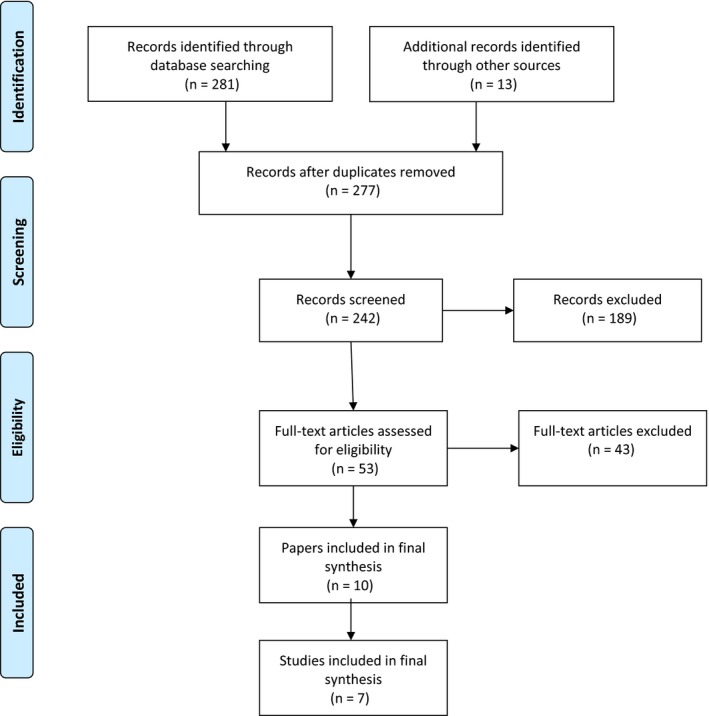
PRISMA diagram

The initial search identified 281 manuscripts. A further 13 articles were found through other sources. After removing duplicates, and screening titles and abstracts, 53 full‐text papers were identified; 43 of these papers were excluded based on the exclusion criteria (see Figure 1). Two members of the research team reviewed papers independently for inclusion in the final analysis.

The final review consisted of 10 papers reporting the outcomes of seven individual studies of multimedia interventions for patients specifically in the acute in‐hospital context. Table 3 summarizes the studies included in the review. Two researchers independently extracted and reviewed the studies and then met to compare and discuss findings. The seven studies all tested multimedia interventions predominately for the purpose of evaluating usability and feasibility in acute care settings; the outcome measures were typically satisfaction ratings.

**Table 3:** Articles included in the review of multimedia interventions to improve patient participation in acute care settings

Author, setting and country	Study design	Purpose	Outcome measures	Intervention	Limitations
Cook et.al^6^, ^68^ Post‐cardiac surgery USA	Quasi‐experimental Post‐test design	1. Test the feasibility of delivering detailed information and acquiring patient‐reported outcome (PRO) measures via iPad_TM_ technology post‐cardiac surgery 2. Test if patient‐reported data were predictive of length of stay or discharge disposition	I‐MOVE mobility scale Length of stay (LOS) Discharge disposition (home or rehabilitation) Modules accessed Patient outcomes reported (pain and mobility scores)	E‐health platform delivered via iPad^TM^ technology Commenced pre‐admission then each day following ICU discharge. Contents: Personalized care plan ‘To do lists’ Self‐assessment tools and reporting capabilities Education specific to surgical procedure Recovery/ discharge planning Early screening for discharge Assessment of mobility	No control group/ comparison group Did not examine participation in recovery outcomes No measure of engagement Reported engagement with the intervention rather than in care No measure of patient outcomes
Dalal et al, and Dykes et al^63^, ^64^ 2 intervention units ‐ medical intensive care unit & oncology unit USA	Quasi‐experimental Post‐intervention test only	To test the enrolment strategy, use and usability of patient tools and patient‐generated message system Pilot test	System usability and user satisfaction survey Number of times system accessed by patient or care giver Number of times messages were sent Number of messages viewed by health team Number of goals or health concerns entered	PCTK (patient‐centred tool kit) that provides patients and care givers ‘tools to participate in plan of care’ Web‐based (computer required) intervention Specific information provided related to test results, medications, team members Ability for patients to interact by sending questions and care goals to health‐care team Used the toolkit for 1‐4 days (MICU) and 5‐10 days (oncology)	No control group/ comparison group Did not examine participation in recovery outcomes Reported engagement with the intervention rather than in care No measure of patient outcomes
Greysen et al^66^ General medical wards USA	Quasi‐experimental Pre‐ and post‐intervention test (pilot)	Prospective study of tablet computers to engage patients in their care and discharge planning through web‐based interactive health education modules and use of personal health record Prospective pilot project to explore inpatient satisfaction with bedside tablets and barriers to usability	Device ownership Health‐related Internet activities Problems with usability Time needed to orientate Overall experience Observation of ability to access information	Web‐based interaction health education modules delivered via tablets Content covered: Medication list Communicating with health‐care team Advanced directives Safety (handwashing & falls prevention) Discharge planning Viewing and modifying appointments	No control group/ comparison group Small sample Did not examine participation in recovery outcomes Reported engagement with the intervention rather than in care No measure of patient outcomes Device only left with patient for 3 to 5 hours
Vardoulakis et al^65^ Emergency department USA	Quasi‐experimental Post‐intervention test only	Feasibility of using a mobile phone device in the emergency department setting. The aim was to present information related to patients’ care plan and care team	Mobile phone feasibility for delivering information Phone usage patterns Patient anxiety Patient empowerment	Presented (via mobile device) a dynamic, interactive report on their progress, care plan, and care team throughout their emergency department stay	No control group/ comparison group Did not examine participation in recovery outcomes Reported engagement with the intervention rather than in care No measure of patient outcomes
Davis et al^25^ General wards UK	Descriptive exploratory Post‐test	To explore patients’ attitudes towards the PINK video, a patient education video aimed at encouraging hospital patients’ involvement in safety‐relevant behaviours	Primary outcome: patient perceptions of relevance, acceptability and how informative the video was and barriers or negative effects of watching Semi‐structured interviews: acceptability, relevance, perceived informative. Attitudes towards participating in recommended behaviours; side‐effects; suggestions for improvement	The PINK video is a short (4 minutes) animated educational video aimed at encouraging patients to be involved in the safety of their care during hospitalization	No control group/ comparison group Did not examine participation in recovery outcomes Reported engagement with the intervention rather than in care No measure of patient outcomes
O’Leary et al^69^ Medical surgical wards USA	Controlled trial ‐ 2 units (medical wards) (one intervention and one control)	To assess the effect of tablet computers with a mobile patient portal application on hospitalized patients' knowledge and activation	(Intervention &Control) interviewed day 2 or 3 to determine knowledge of: Care team members Planned tests/ procedures Medications Activation (PAM)	Frequency of use and satisfaction Patients on the intervention unit were given iPadsTM with the ‘portal application’ for use during their hospitalization	Did have comparative group; however, could have ward‐level confounders Did not examine participation in recovery outcomes Reported engagement with the intervention rather than in care No measure of patient outcomes
Vawdrey et al^67^ Cardiology unit USA	Quasi‐experimental Post‐test design	To evaluate the role tablet computers play in providing information in hospital patient and facilitating communication with health‐care providers Patient satisfaction with, knowledge of, and engagement in their hospital care through semi‐structured interviews.	Semi‐structured interviews: Satisfaction Knowledge Engagement in care 25‐item survey feasibility and acceptability.	Delivered via iPadTM Information about care providers; medications; conditions; tests; procedures	No control group/ comparison group Did not examine participation in recovery outcomes Reported engagement with the intervention rather than in care No measure of patient outcomes.

### 3.2 Usability, feasibility and patient‐related outcomes of using multimedia interventions in acute care

All of the seven studies reviewed reported the usability and feasibility of their interventions in the context of acute care delivery. Table 4 outlines the usability of multimedia in acute care. The degree to which a multimedia intervention is easy to use for patients in the acute care context. These findings suggest that multimedia interventions are both useable and feasible for patient use in the context of acute recovery. Table 5 describes reported outcomes of multimedia interventions in acute care settings, in particular patient‐reported satisfaction, experience and length of stay.

**Table 4:** Studies reporting useability of multimedia interventions in acute care settings

Author	Study design	Purpose	Findings	
			Uptake	Useability
Cook et al^6^, ^68^	Quasi‐experimental Post‐test design	Test the feasibility of delivering detailed information and acquiring patient‐reported outcome (PRO) measures via iPad^TM^ technology post‐cardiac surgery	Patients completed 97.6% of self‐assessment modules	Feasible and effective way to deliver information in post‐operative context There was no measure of patients’ actual knowledge or if their understanding of their recovery increased as a result of the program
Dalal et al and Dykes et al^63^, ^64^	Quasi‐experimental Post‐intervention test only. 2 intervention units ‐ medical intensive care unit (MICU) and oncology unit	Use and usability of patient tools and patient‐generated message system Pilot test	Use of the portal was modest ‐ 66% entered a daily goal; 32% preferences; 7% health concerns; and 64% feedback	Usability scores were moderate to high
Greysen et al^66^	Quasi‐experimental Pre‐ and post‐intervention test (pilot)	Prospective study of tablet computers to engage patients in their care and discharge planning through web‐based interactive health education modules and use of personal health record Prospective pilot project to explore inpatient satisfaction with bedside tablets and barriers to usability	30 patients enrolled in the study 70% accessed PHR 52% communicated with a provider	87% required 30 minutes of education for basic operation Most patients could access modules
Vardoulakis et al^65^	Quasi‐experimental Post‐intervention test only	Feasibility of using a mobile phone device in the emergency department setting. The aim was to present information related to patients’ care plan and care team	25 patients and families (average age 46 years) 22 participants interacted with the phone a total of 10.8 times	Only received 2‐4 minute tutorial Patients reported they liked being in control of the device
Davis et al^25^	Descriptive exploratory. Post‐test.	To explore patients’ attitudes towards the PINK video, a patient education video aimed at encouraging hospital patients’ involvement in safety‐relevant behaviours.	Encouraged ‘willing’ involvement in safety behaviours. 36 participants took part in this study.	Easy to understand. Very informative. Mixed results related to the suitability. Importance was rated highly.
Vawdrey et al^67^	Quasi‐experimental Post‐test design	To evaluate the role tablet computers play in providing information in hospital patient and facilitating communication with health‐care providers Patient satisfaction with, knowledge of, and engagement in their hospital care through semi‐structured interviews	5 patients in cardiac step down unit	Feasible and acceptable way to deliver information to patients in the post‐operation context
O’Leary et al^69^	Controlled trial ‐ 2 units (medical wards) (one intervention and one control)	To assess the effect of tablet computers with a mobile patient portal application on hospitalized patients' knowledge and activation	120 (I) patients given the iPad^TM^. 100 completed the interviews 102 (C) patients interviewed (I) patients younger (*P* = .05)	71% useful information There was no difference between groups in terms of correctly naming a nurse (*P* = .45), awareness of planned tests, procedures or medications

**Table 5:** Reported outcomes of multimedia interventions in acute care settings: Satisfaction and Experience and Length of stay

Author	Study design	Purpose	Findings
Cook et al^6^, ^68^	Quasi‐experimental Post‐test design	Test the feasibility of delivering detailed information and acquiring patient‐reported outcome (PRO) measures via iPad^TM^ technology post‐cardiac surgery	High scores on the mobility scale in early recovery were associated with a reduced LOS Reports of pain had no relationship with LOS
Dalal et al and Dykes et al^63^, ^64^	Quasi‐experimental Post‐intervention test only 2 intervention units ‐ medical intensive care unit (MICU) and oncology unit	Use and usability of patient tools and patient‐generated message system Pilot test	72% were satisfied or extremely satisfied with the tool
Greysen et al^66^	Quasi‐experimental Pre‐ and post‐intervention test (pilot)	Prospective study of tablet computers to engage patients in their care and discharge planning through web‐based interactive health education modules and use of personal health record Prospective pilot project to explore inpatient satisfaction with bedside tablets and barriers to usability	90% satisfied using the tablet
Vardoulakis et al^65^	Quasi‐experimental Post‐intervention test only	Feasibility of using a mobile phone device in the emergency department setting. The aim was to present information related to patients’ care plan and care team	Patients reported they liked being in control of the device
O’Leary et al^69^	Controlled trial ‐ 2 units (medical wards) (one intervention and one control)	To assess the effect of tablet computers with a mobile patient portal application on hospitalized patients' knowledge and activation	76% satisfied

One of the barriers that has been identified in previous research from patients in understanding their care goals and enactment of participation was receiving conflicting or inconsistent information.^59^, ^60^, ^61^, ^62^ To overcome this barrier, Dykes^63^ and Dalal^64^ and colleagues implemented an intervention delivered via interactive web‐based multimedia design, specifically intended to engage hospitalized patients in their plan of care. Outcomes reported included a system usability and satisfaction survey that indicated patients found the system easy to use and were very satisfied (74% satisfied).^63^, ^64^ The most frequently accessed pages via the portal included patient goals, test results, care team members, messages and education regarding tests results and medications.^63^ However, no measure of patients’ ability to understand their plan of care was reported.

Vardoulakis^65^ also confirmed that a multimedia intervention was an acceptable and useable way to deliver consistent and reliable information to patients in acute care. Vardoulakis^65^ utilized a mobile phone application in the emergency department to present information related to patients’ care plan and care team. Patient satisfaction (acceptability) and usability were high amongst the patients and families who engaged with the intervention.^65^ In addition, Greysen^66^ and colleagues found that patients were satisfied with using tablet computers for discharge planning and were able to show that patients engaged with the intervention supporting the notion that usability was possible in this context. This is an important finding that in the fast paced context of an emergency department, patients can utilize these types of platforms to receive information; however, what is not clear is if this information delivered actually leads to improved outcomes or a more engaged patient.

Vawdrey^67^ tested patients’ perceived usefulness and satisfaction with iPad^TM^ technology following cardiac surgery. Participation with the intervention was measured as the number of times the program was accessed by patients.^67^ Whilst the iPad^TM^ was found to be useable and a useful way to deliver information in the acute context, the study outcomes measured did not provide any evidence that patients were more engaged in their care as a function of using the multimedia program. These findings are consistent with previous research where just providing information to patients did not necessarily lead to an increase in participation or have an effect on patient outcomes.^53^, ^54^

Cook et al^68^ investigated whether a multimedia platform would be feasible as a means of collecting patient‐reported outcomes. In this study, 97.6% of patients completed the self‐assessment modules and it was concluded that consumers found the platform useable and that it was a feasible and effective way to deliver information in the post‐operative context.

The majority of reviewed studies were not designed to measure the impact of the intervention on clinical outcomes, and only one study measured patient participation as a function of using multimedia interventions designed to increase patients’ involvement in their care. O'Leary et al^69^ conducted a quasi‐experimental study that included a non‐randomly allocated control group to assess the effect of using an iPad^TM^ with a mobile patient portal application, to improve patients’ knowledge of their health‐care team and their roles, planned tests or procedures, medications and hospitalized patients’ knowledge and activation. O'Leary et al^69^ hypothesized that use of the patient portal would result in greater knowledge of team members’ names and roles, planned tests and procedures, medications, and higher patient activation. The results however were not consistent. Patients who received the intervention were more likely to remember their physicians’ names and roles (*P *=< .001); however, there was no difference between groups in terms of correctly naming a nurse (*P* = .45), or awareness of planned tests (*P* = .33), procedures (*P* = .11) or new medications (*P* = .19) or discontinued medications (*P* = .58). The patient activation measure (PAM) was used to determine differences in the level of activation between groups, but despite there being a trend towards higher activation in the intervention group, no significant difference between groups was revealed. However, it is possible that the study was not sufficiently powered to detect a statically significant difference between groups, as patient activation was not the primary outcome. These findings support that patients’ desire to participate in their care is an important consideration when evaluating interventions designed to improve outcomes.^19^, ^24^ In addition, the power imbalance that exists between clinicians and patient may impact on their capability to participate and this should be taken into consideration when designing and implementing multimedia patient resources.

One indirect measure of patient participation in care and recovery is improvements in clinical outcomes and acute care length of stay.^70^ In one study of an e‐health platform intervention by Cook et al,^68^ patients whose self‐reported mobility scale scores were higher also had a shorter length of stay in hospital compared to standard practice (Table 5). However, it is important to note that there was no objective measure of patient mobility and no concurrent comparison group; nor do Cook et al^68^ claim that the multimedia intervention mediated a change in patient behaviour and subsequently higher self‐reported mobility scores.

In another related study in 2014, Cook et al^6^ tested an e‐health platform as a way to deliver information to older patients after cardiac surgery and found that the majority (98%) indicated they understood the information provided. These responses were however collected using a self‐reported checklist using a dichotomous outcome scale, where patients marked if they did or did not understand the information provided, and no measure of patients’ actual knowledge or understanding of their recovery goals was obtained.

### 3.3 Summary of key findings

All of the studies reviewed reported high patient satisfaction as an outcome of the use of multimedia interventions.^6^, ^67^, ^68^ This is an important finding in terms of ensuring patients are comfortable using this type of intervention in the context of acute care and recovery. Further work is needed using sound methodologies such as randomized controlled trials or quasi‐experimental studies to determine whether multimedia interventions increase patients’ ability to receive and retain information in acute care contexts. In addition, to evaluate if patient participation following the use of these interventions actually lead to better health‐related outcomes.

## 4 DISCUSSION

Patients taking an active role in their own health care have known benefits for patients with chronic illness.^1^, ^2^, ^3^ Finding novel ways to deliver information to patients that is relevant, specific to their needs and unambiguous is a challenge in the context of acute recovery. We know from previous research that patients, on the most part, want to be involved in their decisions made around their care including care transitions.^19^, ^21^, ^31^, ^32^ What this review adds is evidence of the feasibility and usability of multimedia interventions in acute care to provide patients with information relating to their care. There is also some evidence that the usability of multimedia interventions can increase patients’ confidence in their own care‐related knowledge; however without robust research designs, it is unclear if this is due to increased information provision or the use of multimedia platforms to deliver the information.

If we accept that patients do engage with multimedia, what effect does this engagement have on their ability to participate in their care and/or there recovery outcomes? Overall, the studies provide some evidence to suggest that multimedia, as a way to deliver information to patients in the acute care setting, is acceptable to patients and/or caregivers. Further, the time taken to instruct patients to navigate the system, although not always reported, appeared low. Patients showed moderate engagement with the tools; however, the effectiveness of multimedia interventions in increasing patient participation in their care or in improving patient outcomes has not been investigated. In addition, research needs to take into account previous work around patients’ preference for participation,^16^, ^17^, ^18^ just delivering information to patients in a way that is acceptable may not lead to better health outcomes.

A major limitation of the studies reviewed was the quasi‐experimental, post‐test design and lack of a comparative or control group. One exception was O'Leary^7^ who had a control group with similar patient characteristics in both groups that allowed comparisons between those who did and did not receive the intervention. However, the two groups (intervention and control) were allocated to two separate wards in the same hospital^7^ and the structural, process and ward culture characteristics may have differed between wards. Only one of the studies reviewed attempted to investigate whether the interventions had an effect on patient activation, participation or outcomes of care. In addition, the lack of studies provides evidence that patient participation using multimedia interventions is an under‐researched area in acute care. As the studies included in this review were heterogeneous in both the interventions trialled and the outcomes measures, it was not possible to aggregate results or perform meta‐analyses. Another limitation of this narrative systematic review is that ‘grey literature’ was not included. As the use of digital technology and multimedia interventions in health‐care context is a dynamic area of practice innovation, it is acknowledged that evaluations of more recent innovations may not yet be published in the peer‐reviewed literature.

The evidence from this narrative review adds to the growing body of work around the need to engage patients in their own care^4^, ^5^ and the necessity for clinicians to find novel ways to do this in the context of acute care.^51^ The emerging evidence for using multimedia as a platform to do this is encouraging^6^, ^62^, ^63^, ^65^, ^67^, ^68^, ^69^; however, further robust studies are needed to ensure that information delivered in this format to patients leads to better outcomes and improved quality care.

## 5 CONCLUSIONS

There is a worldwide movement to include patients as participants in their own care in the recognition that participation will enhance the quality and safety of the care that patients receive.^71^, ^72^, ^73^ The enactment of patient participation involves a complex interplay between patients’ capability, opportunity and activation.^8^, ^36^, ^74^, ^75^, ^76^ Evidence‐based guidance for facilitating participation in acute care, the implications of patient participation for nursing and health‐care practices and what patient outcomes are likely to be impacted upon is emerging but ill‐defined.^77^ The acute care context presents unique challenges to participation, and it is not clear how patient participation is enacted in this environment, or indeed, if it is possible to implement sustainable interventions to support patient participation. The outcomes of this narrative review demonstrate that using multimedia platforms to deliver information and facilitate patient participation in their care is feasible, and that the useability of these devices by patients is high. As the use of multimedia interventions to improve patient engagement and participation becomes more ubiquitous in health‐care settings, the effectiveness of these interventions needs to be rigorously evaluated.

## CONFLICT OF INTEREST

All authors declare there is no conflict of interest.

## Data Availability

Data sharing is not applicable to this article as no new data were created or analysed in this study.
